# Contrasting Responses to Harvesting and Environmental Drivers of Fast and Slow Life History Species

**DOI:** 10.1371/journal.pone.0148770

**Published:** 2016-02-09

**Authors:** Antoni Quetglas, Lucía Rueda, Diego Alvarez-Berastegui, Beatriz Guijarro, Enric Massutí

**Affiliations:** 1 Instituto Español de Oceanografía, Centre Oceanogràfic de les Balears, Palma de Mallorca, Spain; 2 SOCIB-Balearic Islands Coastal Observing and Forecasting System, Palma de Mallorca, Spain; Technical University of Denmark, DENMARK

## Abstract

According to their main life history traits, organisms can be arranged in a continuum from fast (species with small body size, short lifespan and high fecundity) to slow (species with opposite characteristics). Life history determines the responses of organisms to natural and anthropogenic factors, as slow species are expected to be more sensitive than fast species to perturbations. Owing to their contrasting traits, cephalopods and elasmobranchs are typical examples of fast and slow strategies, respectively. We investigated the responses of these two contrasting strategies to fishing exploitation and environmental conditions (temperature, productivity and depth) using generalized additive models. Our results confirmed the foreseen contrasting responses of cephalopods and elasmobranchs to natural (environment) and anthropogenic (harvesting) influences. Even though a priori foreseen, we did expect neither the clear-cut differential responses between groups nor the homogeneous sensitivity to the same factors within the two taxonomic groups. Apart from depth, which affected both groups equally, cephalopods and elasmobranchs were exclusively affected by environmental conditions and fishing exploitation, respectively. Owing to its short, annual cycle, cephalopods do not have overlapping generations and consequently lack the buffering effects conferred by different age classes observed in multi-aged species such as elasmobranchs. We suggest that cephalopods are sensitive to short-term perturbations, such as seasonal environmental changes, because they lack this buffering effect but they are in turn not influenced by continuous, long-term moderate disturbances such as fishing because of its high population growth and turnover. The contrary would apply to elasmobranchs, whose multi-aged population structure would buffer the seasonal environmental effects, but they would display strong responses to uninterrupted harvesting due to its low population resilience. Besides providing empirical evidence to the theoretically predicted contrasting responses of cephalopods and elasmobranchs to disturbances, our results are useful for the sustainable exploitation of these resources.

## Introduction

In ecology, organisms can be classified according to their main life history traits on a continuum from fast to slow. Fast life history species are characterized by small body size, short lifespan, early reproduction, small offspring size, high fecundity and short generation time; opposite characteristics apply to slow life history species [1,2]. The fast-slow hypothesis is currently the most widely used classification scheme [2] given that the main assumptions of the traditional r-K strategies concept, from which the hypothesis derives, are considered no longer valid [3–5]. Organisms displaying the living fast and dying young strategy are, in general, more productive than those that live more slowly and die older [6,7]. The fast-slow hypothesis has been empirically tested across different taxonomic groups and highlights the interplay among physiology and life-history, ecology and evolution at broad scales [7–9]. Life history determines the responses of organisms to natural (e.g. climate) and anthropogenic (e.g. harvesting) factors, as slow species are expected to be more sensitive than fast species to perturbations [10–13].

In the marine environment, it is well documented that the intense fishing exploitation, with synergistic effects of environmental conditions in some cases, has induced more severe declines in abundance and more noticeable changes in life-history traits of large, slow-growing species than their smaller, faster-growing counterparts (e.g. [11,14–17]). In general, those species growing at slower rates and thus maturing later at greater sizes decreased in abundance compared to their counterparts as a result of harvesting; this entailed concomitant changes in the population structure of these species, such as lower mean individual size and lower maturation size. The fishery-induced truncation of size or age structure can reduce fecundity, elicit declines in harvestable biomass or instability in population growth, and eventually increase the vulnerability of fisheries through reduced resilience [18–21]. Harvested organisms have shown some of the most abrupt trait changes ever observed in wild populations, providing insight for how fast phenotypes can change [10]. Although fishing activity has been identified as the main cause of many marine populations depletions (e.g. [18,20,22]), it is recognised that both abiotic (climate and hydrodynamics) and biotic (trophic resources and predators) environmental variables can also induce intra- and inter-annual oscillations in the population dynamics of some exploited species (e.g. [23–25]).

Owing to their contrasting life histories, cephalopods and elasmobranchs are typical examples of fast and slow strategies, respectively. Cephalopods have short life spans (1.5–2 years at most) and high population growth rates; they have high production, high fecundity and high mortality rates [26]. By contrast, elasmobranchs are long-lived, slow growing and late-maturing, and have low production and low mortality rates [27,28]. As a consequence of these characteristics, cephalopod and elasmobranch populations have high and low resilience respectively.

The sensitivity of cephalopods to natural and human-induced perturbations suggests that they could act as good ecological indicators [29]. Cephalopod populations are more rapidly affected than longer-lived species by external drivers but they are also quicker to recover from perturbations [29–31]. In accordance with the high plasticity of cephalopod populations, substantial changes in biological traits have been reported at different time scales encompassing years [32], seasons [33], weeks [34] and even days [35]. Global cephalopod catch has quadrupled over the last four decades, which seems to be related to the severe decline of many fish stocks [31].

In elasmobranchs, population growth rate and thus recovery potential is, on average, significantly lower (reflecting increased extinction risk) than that of teleosts and terrestrial mammals [36]. The fact that more than half of all chondrichthyan species are predicted to be “Threatened or Near Threatened” according to the IUCN Red List reflects the high vulnerability of elasmobranchs [27,37]. Elasmobranchs typically exhibit rapid declines in catch rates (boom and bust yields), with fisheries collapsing soon after the initiation of exploitation [38]. Although the knowledge on the stock status of elasmobranchs is limited, many populations around the world show dramatic declines or collapses, particularly the large-sized species [27,39].

In this paper, we analyse the responses of fast (cephalopods) and slow (elasmobranchs) life history strategies to fishing exploitation and environmental conditions. According to the contrasting life histories of these two taxonomical groups, the starting hypothesis is that elasmobranch populations will be more highly impacted by harvesting than cephalopod populations owing to the lower resilience of the formers. We further hypothesize that the semelparous, short-lived cephalopods will be, by contrast, more influenced by environmental conditions than the iteroparous, multi-aged elasmobranch populations. Assessing differential responses to harvesting of species with contrasting strategies within an ecological community is essential to manage mixed fisheries under the current Ecosystem Approach to Fisheries [40,41].

## Material and Methods

### Ethic statement

Biological data were obtained from the annual trawl surveys carried out as part of the Mediterranean International Trawl Survey (MEDITS) project. The sampling was performed under repeated international standardized protocol (see [42] for details of the survey methodology). The surveys were conducted across the Spanish territorial waters in the Mediterranean Sea. The research vessels had full permission from national (Fisheries General Secretariat) and international authorities (General Fisheries Commission for the Mediterranean) to sample in territorial and Mediterranean community waters. No approval by an ethics committee was required, as common exploited species were targeted and trawling did not affect endangered or protected species or marine protected areas. Most of the authors participate consistently in the surveys of the MEDITS programme. As most individuals taken by bottom trawl gears arrive dead or in very bad condition on board, it was not necessary to sacrifice them; the most resistant species such as sharks and rays were thrown back to sea alive.

### Sampling and data analysis

**Fig 1:**
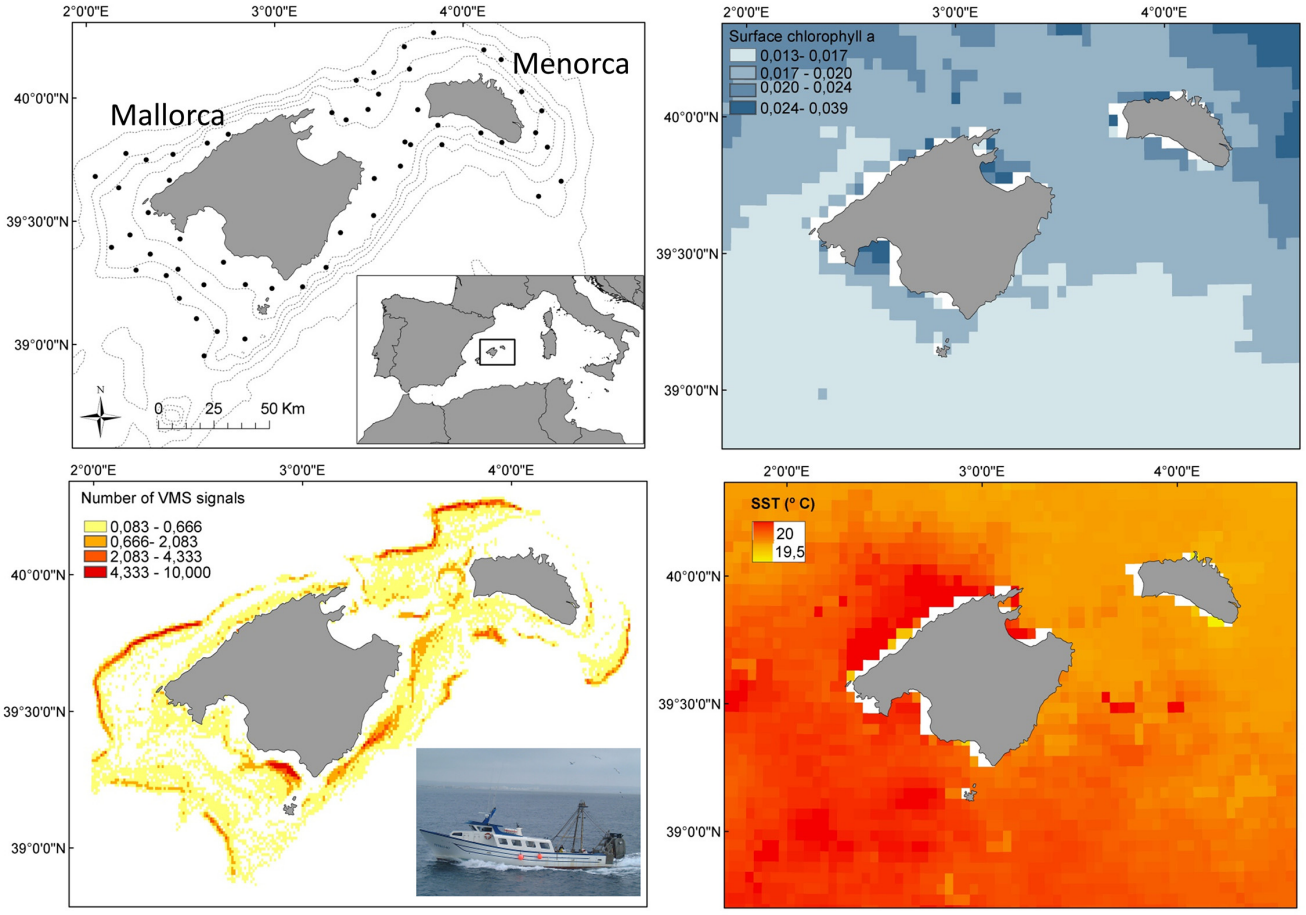
Map of the Balearic Islands (western Mediterranean) showing the sampling stations and the surface chlorophyll-a concentration and sea surface temperature (SST) during the sampling surveys, together with the vessel monitoring system (VMS) records of the bottom trawl fleet operating around the two major islands (Mallorca and Menorca).

Data on cephalopod and elasmobranch abundances were collected during the MEDITS bottom trawl surveys [42] conducted from 2007 to 2012 around the Balearic Islands (western Mediterranean; Fig 1). These surveys are carried out annually at late spring, following a depth stratified random sampling scheme in which a set of approximately 50 stations are sampled. The following four depth strata are considered: A (50–100 m), B (101–200 m), C (201–500 m) and D (501–800 m). The sampling gear is the experimental bottom trawl GOC 73, with a 20 mm mesh codend and average horizontal and vertical net openings of 16.0 and 2.7–3.2 m, respectively. The towing speed is around 2.7–3.0 knots to ensure the best trawl geometry, and the effective trawling duration varies between 20 and 60 min depending on the depth-strata. For each sampling station, the position (latitude, longitude) and depth (m) were taken. The mean density of each studied species was estimated as the total number of individuals by swept area (n Km^-2^). The three most abundant cephalopod (common octopus *Octopus vulgaris*, horned octopus *Eledone cirrhosa* and southern shortfin squid *Illex coindetii*) and elasmobranch (small-spotted catshark *Scyliorhinus canicula*, thornback skate *Raja clavata* and blackmouth shark *Galeus melastomus*) species were selected.

**Fig 2:**
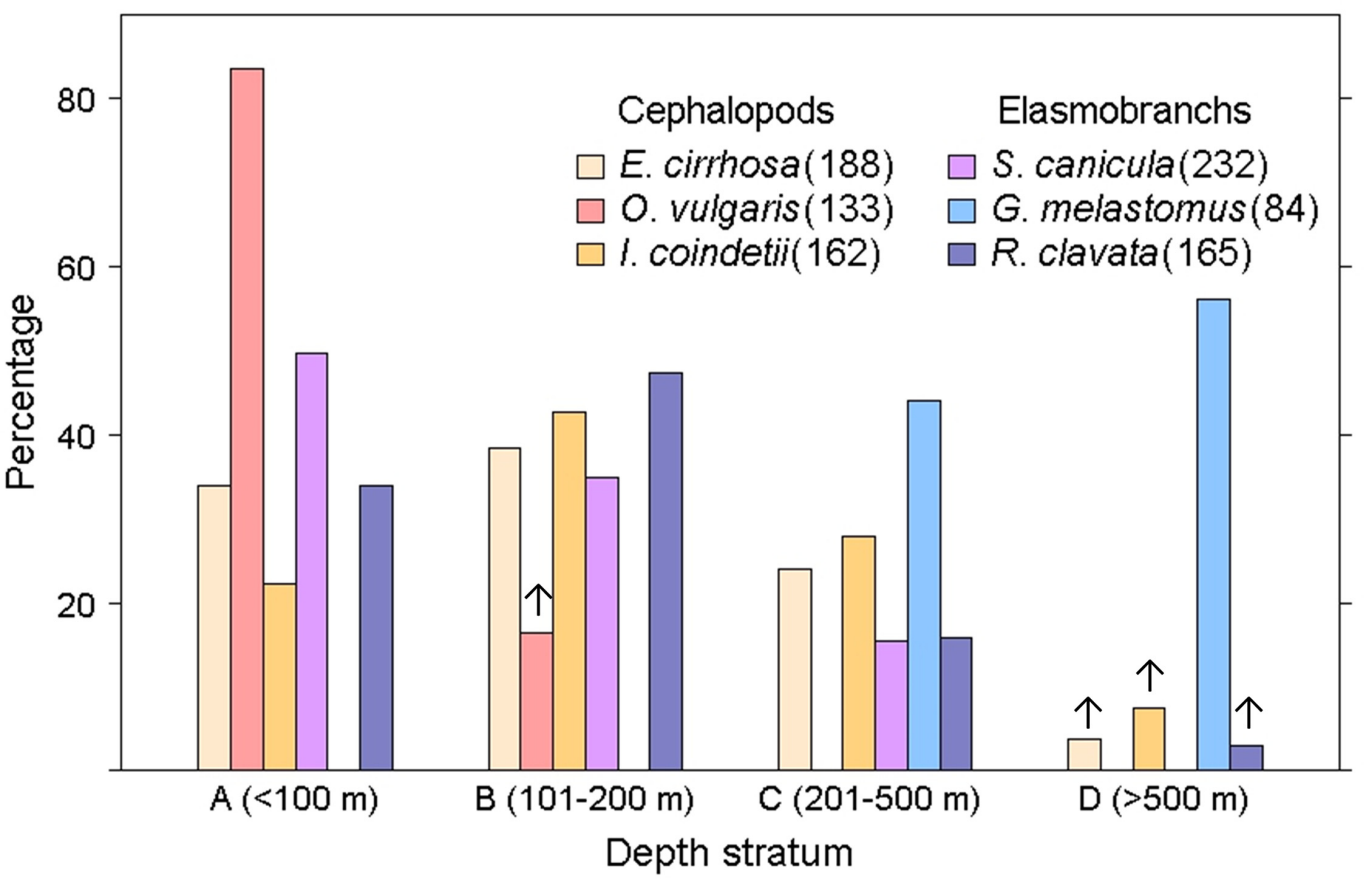
Frequency of occurrence (F%) by depth strata of the fast (cephalopods) and slow (elasmobranchs) life history species analysed. Numbers between brackets are the sampling size and the arrows indicate the datasets removed from the analysis (see Material and methods).

To avoid bias in the results caused by sporadic species occurrences in marginal habitats, only the sampling stations located in the main bathymetric distributional range of each species were considered (Fig 2). In order to show the contrasting life-history traits of cephalopods and elasmobranchs, the main population characteristics of each studied species were summarized from literature (Table 1). All three cephalopods live 2 years at most, while elasmobranchs life-spans range between 7 (*G*. *melastomus*) and 15 (*R*. *clavata*) years. The length of first maturity has been estimated between 10 and 15 cm mantle length for cephalopods and between 40 and 81 cm total length for elasmobranchs. Fecundity is very high in cephalopods (up to hundred thousands oocytes) and hatchlings are planktonic larvae spending a few months on pelagic waters before adopting adult morphology. By contrast, fecundity is very low in elasmobranchs (11–74 eggs) and the eggs give rise to young fish already displaying the main adult characteristics; as a consequence, population resilience in the three investigated elamosbranchs is relatively low (4.5–14 yr; http://www.fishbase.org/).

**Table 1:** Main population traits of the fast (cephalopods) and slow (elasmobranchs) life history species analyzed in this study obtained from the literature: maximum age (in years), maximum individual size, size at first maturity (L_50_) and fecundity. Size and L_50_ (both in cm mantle and total length for cephalopods and elasmobranchs, respectively) are shown for females (F) and males (M) separately.

Taxonomical group	Species	Age (yr)	Size (F/M)	L_50_ (F/M)	Fecundity	Source
Cephalopods	*Octopus vulgaris*	1^a^	27/27^b^	18/10^b^	70,000–650,000^b^	^a^[43]; ^b^[44]
	*Eledone cirrhosa*	1.5^a^	19/15^b^	10/12^b^	550-6500^b^	^a^[45]; ^b^[46]
	*Illex coindetii*	1.5	17/14	15/12	30,000–200,000	[47]
Elasmobranchs	*Scyliorhinus canicula*	12^a^	47/49^b^	40/40^b^	18^b^	^a^[48]; ^b^[49]
	*Raja clavata*	15^a^	110/89^a^	81/67^a^	48/74^b^	^a^[50];^b^[51]
	*Galeus melastomus*	7^a^	64/62^b^	>51/>52^b^	11/30^b^	^a^[52]; ^b^[53]

For each species, symbols “a” and “b” refer to the papers reported on the Source column.

Vessel Monitoring System (VMS) records of bottom trawlers, which is the main fleet targeting the selected species, were used to estimate the fishing effort for the study area from 2007 to 2012. VMS records have been used as a proxy of the fishing exploitation in many previous works (e.g. [54–56]). None of the cephalopods and elasmobranchs analysed here are target species of the bottom trawl fishery, since all of them are taken as a by-catch [57]. This entails that the fishing effort exerted on each of the six species is the same and, in case of finding contrasting responses between groups or among species, these would not be related to contrasting fishing mortalities. Only VMS records with speeds between 1.5 and 5.0 knots, revealing fishing activity [58], were included in the calculations. The sum of records within a radius of 3 km around each sampling station was averaged to account for the specific effect of fishing effort intensity at each station. Due to the clear depth gradient in the area (Fig 1), only the VMS records situated within the same depth strata than the corresponding sampling station were used. As a result of the contrasting life-histories of cephalopods and elasmobranchs, different sensitivities to fishing pressure between groups were expected. As aforementioned, whereas cephalopods are short-lived species dying shortly after reproduction during its first year of life, elasmobranchs are long-lived species with life-spans of several years (see Table 1). Consequently, fishing effort exerted around each station during the previous 3 and 12 months were calculated for cephalopods and elasmobranchs, respectively.

To investigate putative environmental drivers affecting the abundance of both taxonomic groups, we tested the effect of two environmental indicators providing information about the local spatiotemporal changes of sea surface temperature (SST) and chlorophyll-*a* concentration (Chla; mg m^-3^). In order to capture local variations among the different sampling stations, the average Chla within a 9 km radius of the five months previous to the survey (1^st^ January-31^st^ May) was calculated. This period includes the spring bloom, occurring between January and March in the study area [59]. The spatiotemporal average of Chla was computed from weekly means (at 0.05 degrees of spatial resolution) downloaded from the MODIS sensor from the web site of NOAA Coast Watch Program and NASA's Goddard Space Flight Center (http://coastwatch.noaa.gov/). The SST data were obtained from the NCEP/NCAR reanalysis fields *provided by the NOAA/OAR/ESRL PSD* [60]. As previous works reported lagged responses to Chla in fish and cephalopod populations from the western Mediterranean (e.g. [61,62]), two different series of monthly records, spring data contemporary to the survey and data from the previous winter, were used.

In order to estimate the effect of harvesting (VMS records) and environmental conditions (Chla and SST) on the population densities (N km^-2^) of the six selected species, Generalized Additive Models (GAMs) and Generalized Additive Mixed Models (GAMMs) were used. To account for spatial and bathymetric effects, sampling location (latitude, longitude) and depth were also used as covariates. A backward approach, in which only the significant explanatory variables were retained, was used to get the best model. Model selection was based on the Akaike’s information criterion (AIC), which was used as a measure of the goodness of fit as well as the optimal number of model parameters, the best one having the smallest AIC value. Model performance was measured as the proportion of the null deviance explained (DE) or the adjusted regression coefficient (R^2^) when using GAM or GAMM, respectively. Finally, model residuals were checked to fulfil the normality assumption and absence of spatial and temporal autocorrelation. All analyses were implemented with the mgcv library [63] using the R version 3.0.2 (www.R-project.org/).

## Results

**Fig 3:**
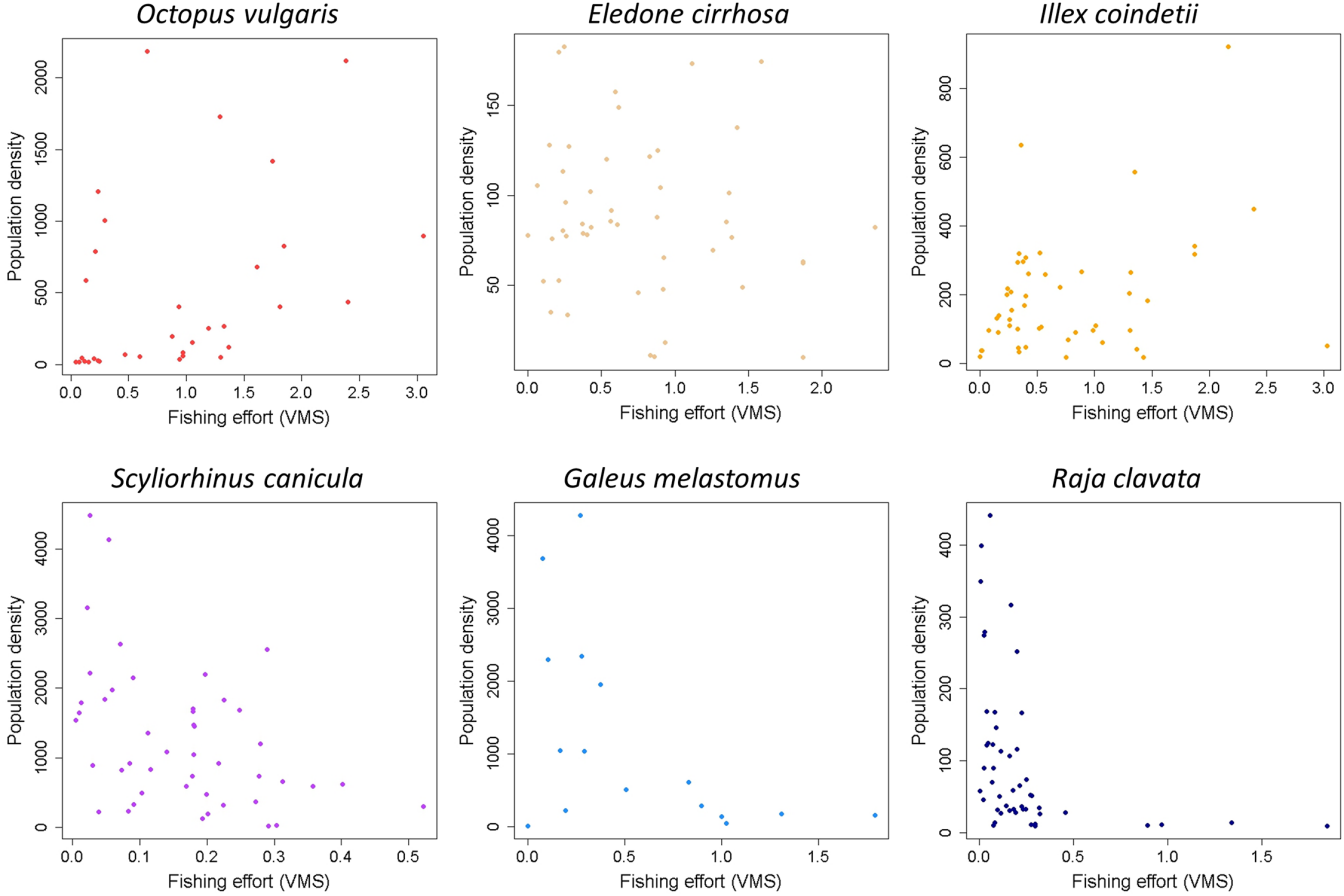
Scatterplots of population densities against fishing effort (VMS, vessel monitoring systems records) for the cephalopods (above) and elasmobranchs (below) species analysed.

The spatial distribution of the sampling stations covered most trawling grounds around the two major Balearic Islands (Mallorca and Menorca) between 50 and 800 m depth, where the commercial fleet works all the year round (Fig 1). The total number of sampling stations analyzed during the study period ranged between 133 (*O*. *vulgaris*) and 188 (*E*. *cirrhosa*) in cephalopods and between 84 (*G*. *melastomus*) and 232 (*S*. *canicula*) in elasmobranchs (Fig 2). There were clear bathymetric differences in the frequency of occurrence of the selected species, being *O*. *vulgaris* and *G*. *melastomus* the species showing the shallowest and deepest distribution, respectively (Fig 2). A first set of exploratory scatterplots representing the densities of each species against the VMS records showed clear contrasting responses between cephalopods and elasmobranchs to the fishing pressure (Fig 3). Whereas the graph did not show any relationship in the former group, the densities of elasmobranchs decreased noticeably with increasing fishing intensity, especially in *R*. *clavata* and *G*. *melastomus*.

The full list of GAM and GAMM models used to test the species densities against fishing effort (VMS), environmental conditions (SST, Chla and depth) and spatial distribution is shown in the Supporting Information; the best model for each species was selected based on the AIC (Table 2). These models gave rise to significant effects in all cases except for *E*. *cirrhosa*, whose mean density in the area was not affected by any of the selected explanatory variables. As we were not able to find a suitable model describing this octopus’ densities using those set of covariates, the following findings refer to the remaining five species.

**Table 2:** Best GAM models obtained for the fast (cephalopods) and slow (elasmobranchs) life history species analysed in this study. Species densities (N km^-2^) were modelled against different covariates (environmental parameters and fishing effort; see Material and methods). Significant covariates, degrees of freedom (DF), goodness of fit (AIC), model performance (DE/R^2^) and sampling size (N) are shown. AIC: Akaike Information Criterion; DE/R^2^: deviance explained (DE, in percentage) or regression coefficient (R^2^) in case of using GAM or GAMM respectively.

Taxonomical group	Species	Covariates	DF	AIC	DE/R^2^	N
Cephalopods	*Octopus vulgaris*	s(SST, k = 4)+s(depth, k = 4)+s(lon, lat, k = 10),random = list(station = ~1)	10	258.8	0.65	108
	*Illex coindetii*	s(SST, k = 4)+s(depth, k = 4),random = list(year = ~1)	7	410.4	0.08	150
Elasmobranchs	*Scyliorhinus canicula*	s(VMS)+s(depth, k = 4)+s(lon, lat, k = 10),correlation = corAR1()	10	630.8	0.38	229
	*Raja clavata*	s(VMS)+s(depth, k = 4)+s(lon, lat, k = 10)	11.4	396.4	44.8	158
	*Galeus melastomus*	s(VMS)+s(depth, k = 4)	5.9	279.3	55.4	83

**Fig 4:**
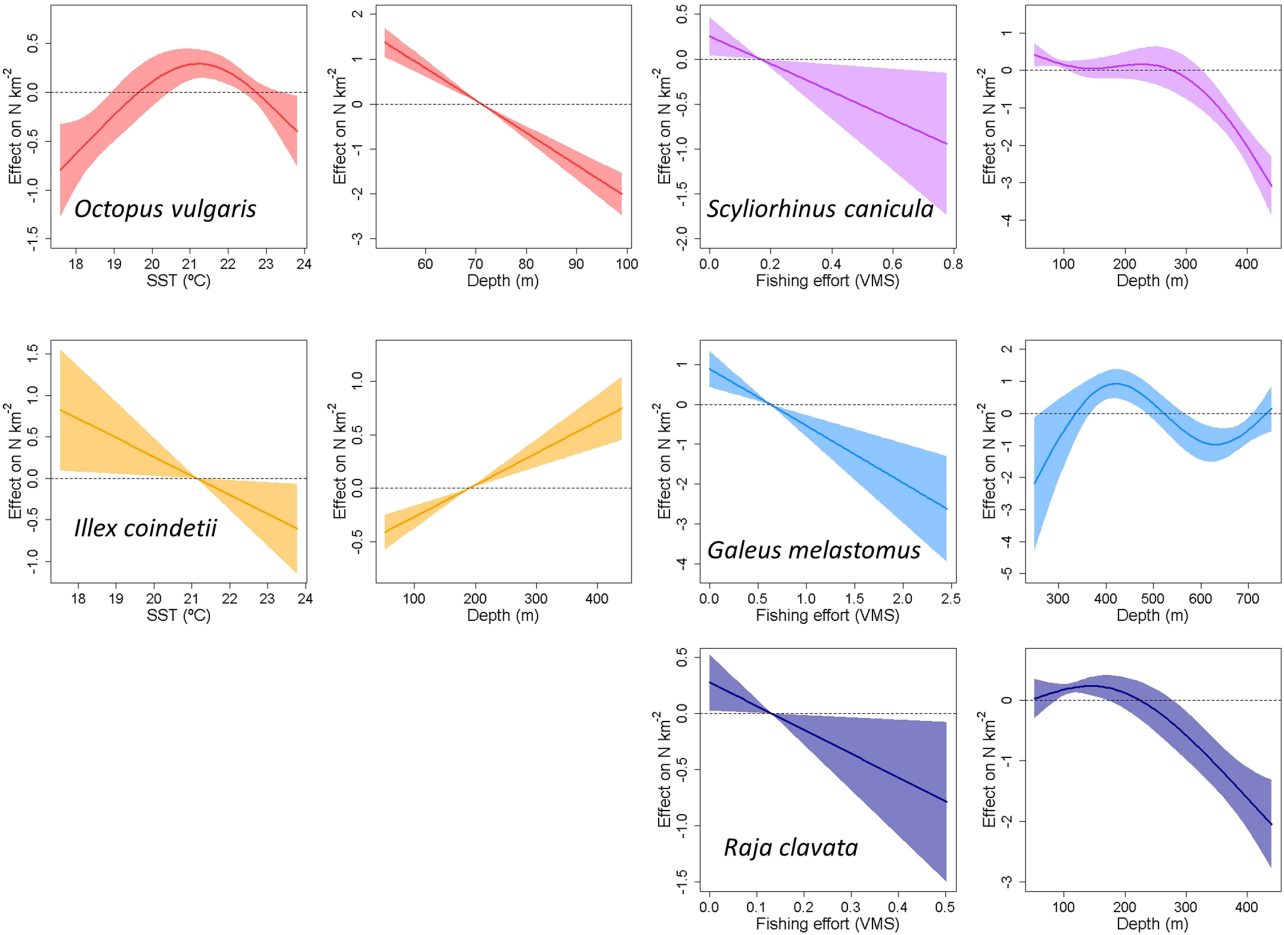
Outputs of the statistically significant generalized additive models (GAM) modelling cephalopod and elasmobranch densities (N km^-2^) against environmental (SST, depth) and fishing effort (VMS) covariates. Model details are in Table 2.

The deviance explained (expressed as proportions), or R^2^ (expressed as percentages), was high in elasmobranchs (*S*. *canicula*, 0.38; *R*. *clavata*, 44.8%; *G*. *melastomus*, 55.4%) as well as in *O*. *vulgaris* (0.65), but much lower in *I*. *coindetii* (0.08). Depth was statistically significant for all species and, interestingly, its effect was lineal in cephalopods but non-lineal in elasmobranchs (Fig 4). Population densities gradually increased and decreased with depth in *I*. *coindetii* and *O*. *vulgaris*, respectively. Whereas densities of *S*. *canicula* and *R*. *clavata* also decreased with depth, *G*. *melastomus* effect was hump-shaped with a maximum at about 400 m. The sampling location was significant for *O*. *vulgaris*, *S*. *canicula* and *R*. *clavata* and it had no effect for *I*. *coindetii* and *G*. *melastomus*

Regarding the remaining factors (fishing effort and environmental variables), the best models differed clearly between groups since cephalopods and elasmobranchs were exclusively driven by environmental variables, namely SST, and fishing exploitation (VMS) respectively. Densities of *I*. *coindetii* decreased linearly with increasing SST, whereas *O*. *vulgaris* densities also showed a hump-shaped trend with a maximum at around 21–22°C. The response of elasmobranchs to fishing exploitation (VMS) was homogeneous, as their population densities decreased linearly with increasing harvesting in all three species.

## Discussion

Our results confirmed the foreseen contrasting responses of fast (cephalopods) and slow (elasmobranchs) life history species to natural (environment) and anthropogenic (harvesting) influences. Even though a priori foreseen, we did expect neither the clear-cut differential responses between groups nor the homogeneous sensitivity to the same factors within the two taxonomic groups. Apart from depth, which affected both groups, cephalopods and elasmobranchs were exclusively affected by environmental conditions (namely SST) and fishing exploitation, respectively. Besides providing empirical evidence to the theoretically predicted contrasting responses of cephalopods and elasmobranchs to disturbances, our study also reveals useful information for the sustainable exploitation of these resources under the current Ecosystem Approach to Fisheries Management (EAFM).

Main life history traits determine population responses to disturbances, as the fast-life history species are more able to withstand them than the slow life history strategists. The fast-slow continuum in life-history not only applies to taxonomic groups with strong differences in life cycles. Strategies can be quite diverse within a taxon or even within populations of the same species [64], whereas distantly related taxa can display similar strategies [65]. Consequently it is inappropriate to generalize a specific strategy to an entire class or family [66], which might explain the lack of significant responses in the horned octopus in contrast with the remaining two cephalopod species analysed. As this octopus was found to be affected by environmental variables in nearby areas [61,62], the lack of significant responses in our study might be related with the highly complex oceanographic conditions at relatively small spatial scales from the western Mediterranean, which has been reported to induce differences in the spatial distribution and life cycles of this species [67,68]. An entire fast-slow continuum also occurs within elasmobranchs [39,69], as they encompass a broad range of life-histories from the small-sized sharks and rays to the giant species (e.g. white shark, whale-shark). Since in our study we analyzed relatively small-sized elasmobranchs situated in the fast corner of this fast-slow continuum, we did not expect the strong responses to harvesting we finally found, as it would have been expected in typical slow living, larger-sized species. Such strong responses to harvesting, however, fully agree with the dramatic declines of these species reported either in our study area [70] and other nearby Mediterranean areas such as the Adriatic Sea [71–74]. To give some figures, elasmobranchs declined by 94.5% over 57 years in the Adriatic [73], with sharks declining more than rays (95.6% vs 87.7%); the small-spotted shark drove most of the patterns (96.2%) and the thornback skate, the most abundant ray in the 1940s, recorded the steepest decline (97.2%). Elasmobranchs are the most endangered group of marine fishes in the Mediterranean, with 31 species assessed as critically endangered, endangered or vulnerable [75].

The observed responses of such comparatively small-sized elasmobranchs agree with the view that body size is not a good indicator of life-history strategies [17,76–79]. In the marine environment, tunas and their relatives constitute good case studies for this view. Time related traits describing the speed of life, rather than size-related traits, better explained the extend and rate of declines and current exploitation status of this taxonomical group [77]. Despite being relatively large (>200 cm), yellowfin tuna is a fast-growing and short lived tropical species that can cope with relatively high fishing mortality rates compared with the similar-sized temperate bluefin tuna. Similar results were obtained when comparing yellowfin tuna with the smaller and lighter elasmobranch blue shark [17]. Blue shark was highly sensitive to low exploitation rates, while yellowfin populations were extremely robust across a wide range of exploitation rates.

In our study, cephalopods were affected by environmental conditions but not by fishing, which tallies with the ecological change in global landings hypothesized by Caddy and Rodhouse [30]. According to these authors, as most coastal and shelf cephalopod fisheries are likely to be fully exploited or overexploited (as is the case in our populations [80]), the current annual fluctuations in their landings are probably largely environmentally-driven. The high sensitivity of cephalopods to environmental conditions is well-know, despite the underlying causes of the links between environment and population dynamics are poorly understood [31]. As a result, cephalopods have been suggested as good ecological indicators of environmental change [29], especially climate change [33], which agrees with the significant sensitivity to sea surface temperature found in our study (but see [81]). In contrast to this view, however, our results indicate that cephalopods would not be good indicators of moderate fishing exploitation. It should be stressed that our results would not imply that cephalopods could cope with any level of harvesting because the fishing exploitation in our study area is moderate compared to nearby areas [82] and responses might be triggered under higher rates. As we hypothesize below, the contrasting responses of cephalopods to environment and harvesting might be related to the fast life history characteristics of this taxonomic group. The lack of response to moderate fishing might also reflect the positive effect that the overfishing of groundfish stocks has had on many cephalopod populations worldwide [30].

Contrary to cephalopods, elasmobranchs were found to be affected by fishing but not by environmental conditions. This is in accordance with the general agreement that the dominant factor in the decline of elasmobranchs has been the fishing exploitation, although probably acting together with synergistic effects of environmental conditions [27,71,72,83,84]. Owing to its slow life history traits (slow growth rate, late maturity, low fecundity), which are more similar to those of large mammals than to other fishes, elasmobranchs are particularly vulnerable to harvesting [27,38,39]. A recent review estimated that one-quarter of elasmobranch species are threatened due to overfishing and that the population depletion is particularly prevalent in the Indo-Pacific Biodiversity Triangle and Mediterranean Sea [27]. The severe decline of large sharks in the Mediterranean during the last two centuries would reflect its long history of intense fishing exploitation [85].

In accordance with previous works (e.g. [11,13,14]), our results indicate that the differential responses of fast (cephalopods) and slow (elasmobranchs) species to harvesting and environmental conditions are governed by their contrasting life history characteristics. Owing to its short, annual cycle, cephalopod populations do not have overlapping generations and consequently lack the buffering effects conferred by different age classes observed in multi-aged species such as elasmobranchs. We suggest that cephalopods are sensitive to short-term perturbations, such as seasonal environmental changes, because they lack this buffering effect but they are in turn not influenced by continuous, long-term disturbances such as moderate fishing, because of its high population growth and turnover. The contrary would apply to elasmobranchs, whose multi-aged population structure would buffer the seasonal environmental effects, but they would display strong responses to uninterrupted harvesting due to its low population resilience. This explanation is in line with Saether et al. [86], when stating that perturbations will affect many age classes in long-lived species, which is likely to result in delayed responses in the dynamics because of covariation in environmental stochasticity producing fluctuations in age structure. In contrast, short-lived species will show far more immediate responses to environmental perturbations, because changes in population size will be caused by demographic variations across most parts of the life cycle.

All cephalopod and elasmobranch species analysed in this study are important by-catch resources from the Mediterranean bottom trawl mixed-fisheries, which take a large number of species having different sensitivities to harvesting. The management of mixed-fisheries constitutes an important challenge, especially in the framework of the Ecosystem Approach to Fisheries, which goes beyond the single-stock strategy, and current approaches based on the maximum sustainable yield (MSY) concept. The MSY has been adopted as the primary management goal by several inter-government fishery organisations (e.g. IWC, ICCAT, IATTC) and has been the cornerstone of the federal fishery policy in the United States for decades [87]. In Europe, the concept has been integrated into the Common Fisheries Policy, with the commitment to maintain or restore their fish stocks to MSY levels by 2020 (EU Regulation N. 1380/2013). The MSY concept, however, has been criticized [88,89] and is especially problematic in the case of mixed-fisheries because it is not possible to simultaneously obtain MSY values for more than one species at a time and alternative approaches are thus required [90–92]. The existence of complex ecological interactions involving the impacts of both the environmental conditions, such as climate change, and the fishing exploitation complicates even further seeking MSY targets at mixed-fisheries or ecosystem levels [90–92]. According to Mackinson et al. [90], taking account of the effect of environmental change and fishing on species dynamics and determining their relative influence is challenging research of vital importance to developing robust long-term fisheries management plans. Our work is in line with this claim since it demonstrates, together with other many studies already reported here, the existence of contrasting sensitivities to natural or anthropogenic disturbances at different taxonomical levels (species, class) that should be taken into account for management purposes and highlights the need for specific strategies adapted to those differential sensitivities.

## Supporting Information

S1 Table List of GAM and GAMM tested in this study to model population density against environmental parameters (SST, Chla, depth) and fishing exploitation (VMS). The degrees of freedom (df), Akaike Information Criteria (AIC), percentage of deviance explained (%DE) or regression coefficient (R^2^) and the number of samples (n) are also shown. The best model number is highlighted with an asterisk.
(DOCX)
