# Supplementary material for: Contrasting Responses to Harvesting and Environmental Drivers of Fast and Slow Life History Species
Source: PLoS One. 2016 Feb 9;11(2):e0148770. doi: 10.1371/journal.pone.0148770 (PMC4747561; doi:10.1371/journal.pone.0148770)
Supplement: S1 Table — The degrees of freedom (df), Akaike Information Criteria (AIC), percentage of deviance explained (%DE) or regression coefficient (R2) and the number of samples (n) are also shown. The best model number is highlighted with an asterisk. (DOCX) [file pone.0148770.s001.docx]

| **Species** | **Model number** | **Model structure** | **df** | **AIC** | **% DE/ R^2^** | **n** |
| --- | --- | --- | --- | --- | --- | --- |
| G. melastomus | 0 | log(N_km2)~1 | 2 | 338.4529 | 0 | 83 |
|  | 1 | log(N_km2)~s(Chla_01to05)+s(VMS_d12m)+s(sst_mean0,k=4)+s(depth,k=4)+s(lon,lat,k=10) | 16.511569 | 278.5868 | 65.7 | 83 |
|  | 2 | log(N_km2)~s(Chla_01to05)+s(VMS_d12m)+s(sst_mean0,k=4)+estratum+s(lon,lat,k=10) | 15.344745 | 279.5528 | 64.3 | 83 |
|  | 3 | log(N_km2)~s(Chla_01to05)+s(VMS_d12m)+s(sst_mean0,k=4)+s(depth,k=4) | 8.957056 | 279.3567 | 58.5 | 83 |
|  | 4 | log(N_km2)~s(Chla_01to05)+s(VMS_d12m)+s(sst_mean0,k=4)+estratum | 8.576643 | 288.3059 | 53.4 | 83 |
|  | *5** | *log(N_km2)~s(VMS_d12m)+s(depth,k=4)* | *5.918561* | *279.3273* | *55.4* | *83* |
|  | 6 | log(N_km2)~s(VMS_d12m)+s(lon,lat,k=10) | 11.739119 | 281.2448 | 60.3 | 83 |
|  | 7 | log(N_km2)~s(VMS_d12m)+estratum | 4.478024 | 289.2544 | 47.9 | 83 |
|  | 8 | log(N_km2)~s(VMS_d12m)+s(depth,k=4),correlation=corAR1() | 7 | 293.0793 | 0.531 | 83 |
|  | 9 | log(N_km2)~s(VMS_d12m)+s(depth,k=4),random=list(station=~1) | 7 | 292.3219 | 0.531 | 83 |
| S. canicula | 0 | log(N_km2) ~ 1 | 2 | 763.5978 | 0 | 229 |
|  | 1 | log(N_km2) ~ s(Chla_01to05) + s(VMS_d12m) + s(sst_mean0, k = 4) + s(depth, k = 4) + s(lon, lat, k = 10) | 15.268943 | 650.8771 | 45.6 | 229 |
|  | 2 | log(N_km2) ~ s(Chla_01to05) + s(VMS_d12m) + s(sst_mean0, k = 4) + estratum + s(lon, lat, k = 10) | 15.432201 | 687.4126 | 36.2 | 229 |
|  | 3 | log(N_km2) ~ s(VMS_d12m) + s(depth, k = 4) + s(lon, lat, k = 10) | 13.427492 | 649.0585 | 45.1 | 229 |
|  | 4 | log(N_km2) ~ s(VMS_d12m) + estratum + s(lon, lat, k = 10) | 12.883107 | 687.3021 | 34.8 | 229 |
|  | 5 | log(N_km2) ~ s(VMS_d12m) + s(depth, k = 4) | 5.910389 | 675.9854 | 34.1 | 229 |
|  | 6 | log(N_km2) ~ s(VMS_d12m) + estratum | 6.366196 | 706.8863 | 24.9 | 229 |
|  | *7** | *log(N_km2)~s(VMS_d12m)+s(depth,k=4)+s(lon,lat,k=10),correlation=corAR1()* | *10* | *630.8231* | *0.381* | 229 |
|  | 8 | log(N_km2)~s(VMS_d12m)+s(depth,k=4)+s(lon,lat,k=10),random=list(station=~1) | 10 | 651.1708 | 0.39 | 229 |
|  | 9 | log(N_km2) ~ s(VMS_d12m) + s(depth, k = 4) + s(lon, lat, k = 10),correlation=corGaus() | 10 | 633.4318 | 0.382 | 229 |
|  | 10 | log(N_km2)~s(VMS_d12m)+s(depth,k=4),random=list(station=~1) | 5.910389 | 675.9854 | 34.1 | 229 |
| R. clavata | 0 | log(N_km2) ~ 1 | 2 | 471.6715 | 0 | 158 |
|  | 1 | log(N_km2)~s(Chla_01to05)+s(VMS_d12m)+s(sst_mean0,k=4)+s(depth,k=4)+s(lon,lat,k=10) | 15.488261 | 394.91 | 48.1 | 158 |
|  | 2 | log(N_km2)~s(Chla_01to05)+s(VMS_d12m)+s(sst_mean0,k=4)+estratum+s(lon,lat,k=10) | 16.039065 | 410.6694 | 43.1 | 158 |
|  | *3** | *log(N_km2)~s(VMS_d12m)+s(depth,k=4)+s(lon,lat,k=10)* | *11.387175* | *396.4686* | *44.8* | *158* |
|  | 4 | log(N_km2)~s(Chla_01to05)+s(VMS_d12m)+estratum+s(lon,lat,k=10) | 14.809544 | 410.247 | 42.4 | 158 |
|  | 5 | log(N_km2)~s(VMS_d12m)+s(depth,k=4) | 5.217432 | 416.1337 | 32.4 | 158 |
|  | 6 | log(N_km2)~s(VMS_d12m)+s(depth,k=4)+s(lon,lat,k=10),correlation=corAR1() | 10 | 405.2888 | 0.411 | 158 |
|  | 7 | log(N_km2)~s(VMS_d12m)+s(depth,k=4)+s(lon,lat,k=10),random=list(station=~1) | 10 | 396.3264 | 0.392 | 158 |
| E. cirrhosa | 0 | log(N_km2) ~ 1 | 2 | 440.4878 | 0 | 178 |
|  | 1 | log(N_km2)~s(Chla_01to05)+s(VMS_s6m)+s(sst_mean0,k=4)+s(depth,k=4)+s(lon,lat,k=10) | 10.157806 | 445.823 | 5.98 | 178 |
|  | 2 | log(N_km2)~as.factor(estratum.1) | 4 | 442.7188 | 0.989 | 178 |
|  | 3 | log(N_km2)~s(depth,k=4) | 3.585349 | 439.3243 | 2.41 | 178 |
|  | 4 | log(N_km2)~s(Chla_01to05) | 3 | 442.4851 | 0.0015 | 178 |
|  | 5 | log(N_km2)~s(VMS_s6m) | 3 | 442.1188 | 0.207 | 178 |
|  | 6 | log(N_km2)~s(media_s3m) | 3 | 442.475 | 0.00716 | 178 |
|  | 7 | log(N_km2)~s(sst_mean0) | 3.247582 | 442.3592 | 0.35 | 178 |
|  | 8 | log(N_km2)~s(lon,lat,k=10) | 5.537475 | 440.5393 | 3.87 | 178 |
| I. coindetii | 0 | log(N_km2)~1 | 2 | 489.1228 | 0 | 150 |
|  | 1 | log(N_km2)~s(Chla_01to05)+s(VMS_s6m)+s(sst_mean0,k=4)+s(depth,k=4)+s(lon,lat,k=10) | 11.462175 | 460.117 | 27.4 | 150 |
|  | 2 | log(N_km2)~s(Chla_01to05)+s(VMS_s6m)+s(sst_mean0,k=4)+estratum.1+s(lon,lat,k=10) | 12.990621 | 464.7845 | 26.6 | 150 |
|  | 3 | log(N_km2)~s(Chla_01to05)+s(media_s3m)+s(sst_mean0,k=4)+s(depth,k=4)+s(lon,lat,k=10) | 11.065752 | 459.8941 | 27.1 | 150 |
|  | 4 | log(N_km2)~s(sst_mean0,k=4)+s(depth,k=4) | 6.152325 | 457.5411 | 23.3 | 150 |
|  | 5 | log(N_km2)~s(sst_mean0,k=4)+s(depth,k=4),random=list(station=~1) | 7 | 471.7798 | 0.209 | 150 |
|  | 6 | log(N_km2)~s(sst_mean0,k=4)+s(media_s3m)+s(depth,k=4),random=list(year=~1) | 9 | 414.1643 | 0.0933 | 150 |
|  | 7 | log(N_km2)~s(sst_mean0,k=4)+s(media_s3m)+estratum.1,random=list(year=~1) | 9 | 417.1731 | 0.0542 | 150 |
|  | *8** | *log(N_km2)~s(sst_mean0,k=4)+s(depth,k=4),random=list(year=~1)* | *7* | *410.4319* | *0.0849* | *150* |
| O. vulgaris | 0 | log(N_km2)~1 | 2 | 365.332 | 0 | 108 |
|  | 1 | log(N_km2)~s(Chla_01to05)+s(VMS_s6m)+s(sst_mean0,k=4)+s(depth,k=4)+s(lon,lat,k=10) | 10.053846 | 257.1335 | 68.4 | 108 |
|  | 2 | log(N_km2)~s(sst_mean0,k=4)+s(depth,k=4)+s(lon,lat,k=10) | 7.517251 | 254.6628 | 67.6 | 108 |
|  | 3 | log(N_km2)~s(Chla_01to05)+s(VMS_s6m)+s(sst_mean0,k=4)+s(lon,lat,k=10) | 14.697004 | 323.7799 | 46.2 | 108 |
|  | 4 | log(N_km2)~s(Chla_01to05)+s(VMS_s6m)+s(lon,lat,k=10) | 13.06589 | 323.7949 | 44.5 | 108 |
|  | 5 | log(N_km2)~s(Chla_01to05)+s(VMS_s6m)+s(sst_mean0,k=4)+s(lon,lat,k=10) | 14.697004 | 323.7799 | 46.2 | 108 |
|  | 6 | log(N_km2)~s(sst_mean0,k=4)+s(depth,k=4)+s(lon,lat,k=10),correlation=corAR1() | 10 | -223.3237 | -0.0276 | 108 |
|  | *7** | *log(N_km2)~s(sst_mean0,k=4)+s(depth,k=4)+s(lon,lat,k=10),random=list(station=~1)* | *10* | *258.8006* | *0.655* | 108 |
|  | 8 | log(N_km2)~s(sst_mean0,k=4)+s(depth,k=4)+s(lon,lat,k=10),random=list(year=~1) | 10 | 267.221 | 0.656 | 108 |
